# Self-guided Cognitive Behavioral Therapy Apps for Depression: Systematic Assessment of Features, Functionality, and Congruence With Evidence

**DOI:** 10.2196/27619

**Published:** 2021-07-30

**Authors:** Laura Martinengo, Anne-Claire Stona, Konstadina Griva, Paola Dazzan, Carmine Maria Pariante, Florian von Wangenheim, Josip Car

**Affiliations:** 1 Centre for Population Health Sciences Lee Kong Chian School of Medicine Nanyang Technological University Singapore Singapore Singapore; 2 Department of Psychological Medicine Institute of Psychiatry, Psychology and Neuroscience King’s College London London United Kingdom; 3 National Institute for Health Research Biomedical Research Centre at South London and Maudsley NHS Foundation Trust London United Kingdom; 4 Institute of Psychiatry, Psychology & Neuroscience (IoPPN) King's College London London United Kingdom; 5 Professor of Technology Marketing Department of Management, Technology & Economics ETH Zurich Zurich Switzerland; 6 Department of Primary Care and Public Health School of Public Health Imperial College London London United Kingdom

**Keywords:** cognitive behavioral therapy, CBT, depression, mobile applications, apps, telemedicine, mHealth, self-guided CBT-based apps, self-management, mobile phone

## Abstract

**Background:**

Mental health disorders affect 1 in 10 people globally, of whom approximately 300 million are affected by depression. At least half of the people affected by depression remain untreated. Although cognitive behavioral therapy (CBT) is an effective treatment, access to mental health specialists, habitually challenging, has worsened because of the COVID-19 pandemic. Internet-based CBT is an effective and feasible strategy to increase access to treatment for people with depression. Mental health apps may further assist in facilitating self-management for people affected by depression; however, accessing the correct app may be cumbersome given the large number and wide variety of apps offered by public app marketplaces.

**Objective:**

This study aims to systematically assess the features, functionality, data security, and congruence with evidence of self-guided CBT-based apps targeting users affected by depression that are available in major app stores.

**Methods:**

We conducted a systematic assessment of self-guided CBT-based apps available in Google Play and the Apple App Store. Apps launched or updated since August 2018 were identified through a systematic search in the 42matters database using CBT-related terms. Apps meeting the inclusion criteria were downloaded and assessed using a Samsung Galaxy J7 Pro (Android 9) and iPhone 7 (iOS 13.3.1). Apps were appraised using a 182-question checklist developed by the research team, assessing their general characteristics, technical aspects and quality assurance, and CBT-related features, including 6 evidence-based CBT techniques (ie, psychoeducation, behavioral activation, cognitive restructuring, problem solving, relaxation, and exposure for comorbid anxiety) as informed by a CBT manual, CBT competence framework, and a literature review of internet-based CBT clinical trial protocols. The results were reported as a narrative review using descriptive statistics.

**Results:**

The initial search yielded 3006 apps, of which 98 met the inclusion criteria and were systematically assessed. There were 20 well-being apps; 65 mental health apps, targeting two or more common mental health disorders, including depression; and 13 depression apps. A total of 28 apps offered at least four evidence-based CBT techniques, particularly depression apps. Cognitive restructuring was the most common technique, offered by 79% (77/98) of the apps. Only one-third of the apps offered suicide risk management resources, whereas 17% (17/98) of the apps offered COVID-19–related information. Although most apps included a privacy policy, only a third of the apps presented it before account creation. In total, 82% (74/90) of privacy policies stated sharing data with third-party service providers. Half of the app development teams included academic institutions or health care providers.

**Conclusions:**

Only a few self-guided CBT-based apps offer comprehensive CBT programs or suicide risk management resources. Sharing of users’ data is widespread, highlighting shortcomings in health app market governance. To fulfill their potential, self-guided CBT-based apps should follow evidence-based clinical guidelines, be patient centered, and enhance users’ data security.

## Introduction

### Background

Mental health disorders affect 1 in 10 people globally [1], of which approximately 300 million are affected by depression [2]. At least half of the affected people remain untreated [3-5] because of many reasons [6-8], including low perceived need for treatment and self-reliance [7,8], stigma [9], difficulty accessing specialist care [10], fragmentation of the mental health care system [11], and high costs of treatment [12]. Access to psychiatrists and clinical psychologists is challenging, particularly in low- and middle-income countries [13] and for people of lower socioeconomic status, poorer health, and rural and hard-to-reach communities in high-income economies [14,15]. Since the onset of the COVID-19 pandemic, these inadequacies have been exacerbated by physical isolation, and fear and uncertainty [16,17] led to a sudden increase in mental health concerns [18].

Cognitive behavioral therapy (CBT), one of the most widely used and researched forms of psychotherapy [19], is effective in treating a wide range of mental disorders, including depression [20-22]. CBT is a “structured, time-limited, present-focused” [23] therapy that promotes behavioral adaptation by coaching users to use a variety of tailored cognitive and behavioral techniques [24]. Traditional face-to-face CBT is costly, time-consuming, and subject to the availability of trained providers [10], limiting accessibility. To increase access to therapy, a number of internet-based CBT (iCBT) programs [25-27] offer an acceptable and effective alternative [28-30]. Therapist-guided iCBT has been shown to be as effective as face-to-face sessions [28], whereas self-guided interventions, albeit effective, are not at par with guided web-based programs [30-32].

Over the last decade, there has been increasing interest in using smartphone apps to support mental health disorder management and well-being [33], which has been further intensified since the COVID-19 pandemic [34,35]. Recent reports indicate that nearly 320,000 health apps [36] are available in major app stores, of which more than 10,000 are mental health apps [37]. Smartphone penetration is increasing worldwide, particularly in high-income countries [38], making apps a useful means to improve access to care for people unable or unwilling to consult a health care provider by offering interventions to be used at the time and place of the user’s convenience at low cost [39,40]. Apps can improve patient activation and disease self-management by increasing access to information and facilitating communication with health care providers and peers [41]. Two recent reviews, comprising one systematic review and one umbrella review [42,43], concluded that apps were effective in improving users’ emotional symptoms, including depression, although effect sizes varied according to the intervention and comparator groups, as well as the study quality. However, several shortcomings of mental health apps have been repeatedly described, including substantial dropout rates [44], mishandling of users’ personal health information [45], poor app credibility, and lack of content personalization [41]. In addition, most health apps available in major app stores are not evidence based and have not been validated in clinical trials or approved by regulatory agencies [37,46], underlining concerns about the effectiveness and safety of publicly available apps.

Previous work on self-guided CBT apps for depression noted only a few apps offering some evidence-based techniques, with limited user engagement features and a dominant focus solely on depression [47,48]. However, people with depression searching for an app will often retrieve a much wider variety of apps, including apps targeting several mental health disorders or those aimed at improving general well-being for healthy individuals. Therefore, we considered it important to conduct an updated assessment of self-guided CBT-based apps available in Google Play and Apple App Store encompassing well-being, general mental health, and depression apps to more closely align with real-life users’ experience.

### Objective

This study aims to systematically assess features, functionality, and congruence with evidence of self-guided CBT-based apps available in major app stores, targeting users affected by depression.

## Methods

### Overview

We developed a rigorous systematic assessment based on systematic literature review methodology, as used in several other studies [49-52]. The process included a systematic search for apps, two-step selection, and assessment following an exhaustive set of criteria developed by the research team.

### Development of Assessment Criteria

The assessment criteria aimed to provide an in-depth and holistic analysis of the apps, including clinical and technical characteristics. CBT-related features constitute a fundamental aspect of the overall appraisal, particularly the assessed apps’ content compliance with evidence-based practice, as all health apps should ideally be based on sound evidence. Technical characteristics were included in the assessment if they were available in the app, app store description, or associated website and were not dependent on the assessor’s subjective opinion or required access to the app’s back-end data. The form was divided into three sections:

1. *General characteristics* extracted from the app store description, including developer, platform, app version number, category and ratings, number of downloads, cost, country of origin, languages featured in the app, target group, scope of the app, and therapy modalities offered by the app.
2. *CBT-related features*, based on *Cognitive Behavior Therapy: Basics and Beyond* by Judith Beck [53], a CBT competence framework [54] developed by the Improving Access to Psychological Therapies program in the United Kingdom, and a literature review of iCBT clinical trial protocols to identify distinct characteristics relevant to digital interventions. They comprised the following (Multimedia Appendix 1):
  - *Evidence-based CBT techniques*, as described in the reference sources mentioned earlier, are routinely used in face-to-face practice: *psychoeducation*, about depression and CBT;*behavioral activation (BA)*, including activity and task scheduling, suggestion of pleasurable activities, and monitoring of completed activities; *cognitive restructuring*, including assessment of automatic thoughts and core beliefs using thought records and completing behavioral experiments to challenge automatic thoughts and core beliefs; and *other techniques* frequently used in CBT, including *problem solving*, *relaxation*, and *exposure techniques *for comorbid anxiety.
  - *Procedures related to the structure of face-to-face CBT sessions*: CBT sessions are highly structured and offer users strategies to cope after the end of therapy. We assessed whether relevant components of CBT sessions were offered, including structured, modular sessions, mood monitoring, suicide risk assessment, goal setting, homework assignment, *therapeutic alliance* [55], and coping strategies after completing the program. Following Tremain et al [55], we conceptualized the *therapeutic alliance* for self-guided interventions as strategies that encourage users’ engagement with the app and adherence to assigned tasks, such as gamification, notifications, and reminders.
  - *Other information*, including access to professional advice for distressed users, information related to the mental health impact of the COVID-19 pandemic as a proxy for continuous improvement of app content, and other techniques not mentioned elsewhere.
3. *Technical aspects and quality assurance of the app*, based on an assessment framework developed by our center [51,52], comprising ease-of-use, app credibility, presence of advertisements, privacy and security safeguards, including thoroughness of the privacy policy, privacy settings, and authentication to access the app content, among others. App credibility included appropriately referenced app content, qualifications of the app development team, presence of *information does not replace provider’s advice* disclaimers, and published evidence of app effectiveness, assessed by searching PubMed for publications using the app name as keyword.

### App Selection

#### App Search

A systematic search for apps available in the Apple App Store and Google Play Store was performed on February 19, 2020, in 42matters [56], a proprietary database, using the search terms *cognitive behavioral therapy*, *cognitive behavioural therapy*, *cognitive therapy*, *CBT*, *behavioral therapy*, *behavioural therapy*, *behavioral activation*, *behavioural activation*, *online therapy*, *psychotherapy*, *counselling,* and *talking therapy*. A total of 4 app store categories were included: education, health and fitness, lifestyle, and medical. For apps available in only one app store, a web search was performed to look for the other version, and if available, it was downloaded and assessed.

#### Eligibility Criteria

We included apps described as based on CBT, exclusively or associated to other psychotherapeutic modalities; offering CBT-based activities, exclusively or associated to other psychotherapeutic modalities; aiming to improve mood and well-being for people with low mood or depression and targeting depression alone or associated with another mental disorder; uploaded or updated from August 1, 2018, as regular updates of an app seem to directly affect app quality [57]; free, freemium (app is free to download but requires payment to activate extra features), or paid; available for download in the Apple App Store or Google Play Store; and in English.

We excluded apps offering CBT for other mental health disorders (ie, standalone anxiety, insomnia, posttraumatic stress disorder, etc); offering non–CBT-based mood recording or journaling, as referred to in the app store description; offering health care provider– or counselor-guided CBT modules, targeting health care providers or caregivers of a person affected by depression; offering teleconsultation services with physicians, psychologists, counselors, or other health care providers; removed from the app stores at the time of download, requiring a sign-up code provided by an institution, or inaccessible after two attempts due to technical problems; and in a language other than English.

### App Assessment

App selection followed a systematic, two-step process. We first screened the app title and app store description from the 42matters search output and downloaded all apps included in the first step to assess eligibility. All assessments were performed using a Samsung Galaxy J7 Pro (Android 9) and iPhone 7 (iOS 13.3.1). If apps were available in both app stores, both versions were assessed to account for any difference in functionalities, and each version was included and counted separately. Individual apps belonging to a suite of related apps were combined and counted as one app.

Eligible apps were then assessed by the researcher (a medical doctor) for all available CBT techniques. If the app presented a modular intervention, it was used repeatedly to complete all modules. For consistency of assessments, a user persona was developed outlining demographic information, personal and medical history, responses to assessment questionnaires, and opening statements for conversational agent dialogs.

### Data Analysis

Descriptive statistics were used to analyze the data. The study results were tabulated and reported as a narrative synthesis. Data extraction and analysis were performed using Microsoft Excel.

## Results

### Overview

**Figure 1:**
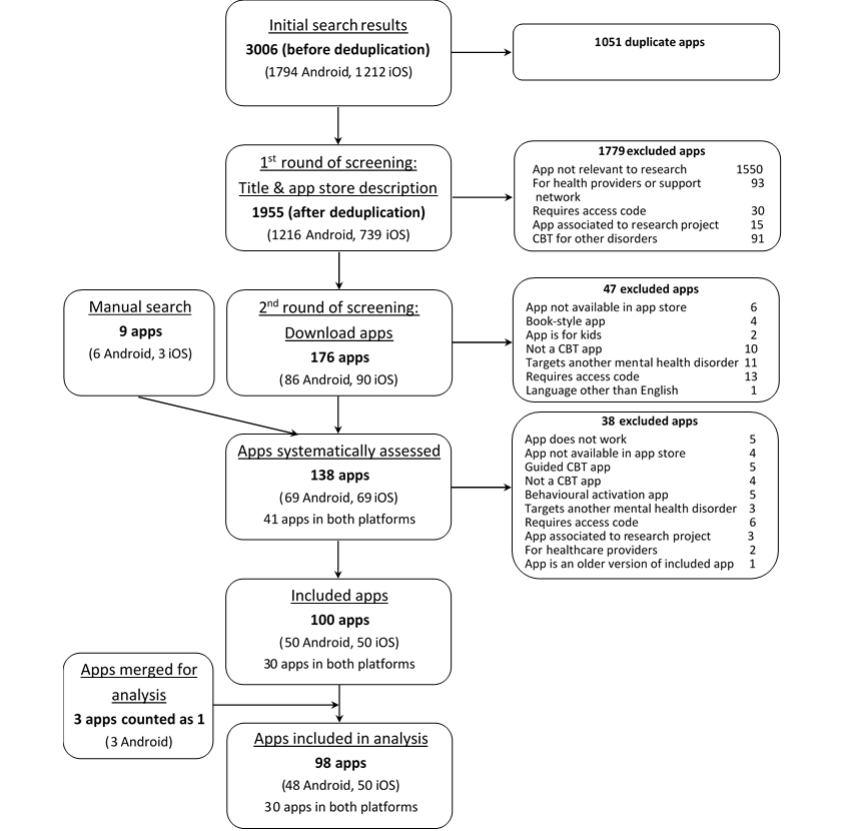
App selection flowchart. CBT: cognitive behavioral therapy.

The keyword search retrieved 1955 results after duplicates were removed, of which 140 apps were downloaded and 100 apps were included in this analysis. A total of 3 apps (ie, eGuru Depression [58], eGuru Mood Diary [59], and eGuru Thought Diary [60]) belonged to a suite of related apps and were analyzed together as one app. Therefore, 98 apps were finally included in the analysis. The app search and selection processes are summarized in Figure 1. Multimedia Appendix 2 presents the list of assessed apps and a summary of CBT-related features.

### General Characteristics of Apps

Table 1 summarizes the characteristics of the included apps. Apps were grouped into three distinct categories: (1) *well-being apps*, providing CBT-based activities to improve users’ general well-being; (2) *mental health apps*, offering self-guided activities to manage two or more common mental health disorders, including depression; and (3) *depression apps* offering self-guided CBT exclusively for depression.

There were 48 Android apps and 50 iOS apps. A total of 30 apps were available on both platforms, 18 were Android-only apps and 20 were iOS-only apps. A total of 74% (73/98) of apps belonged to the health and fitness app store category, whereas 19% (19/98) of apps were listed as medical apps. A total of 27% (13/48) of Android apps were downloaded more than 100,000 times, including 11 mental health apps, 1 depression app, and 1 well-being app. A total of 4 mental health apps (ie, Moodpath [61], Sanvello [62], Wysa [63], and Youper [64]) and 1 well-being app (ie, Reflectly [65]) were downloaded more than 1,000,000 times. Three-quarter (n=73) of the apps had users’ ratings above 3.5 on a 1-5 scale and 42% (41/98) of apps had ratings above 4.5. Apps were developed in 16 different countries (Table 2).

**Table 1:** General characteristics of the included apps (N=98).

Feature			App category, n (%)							Total (N=98), n (%)
			Well-being (n=20)		Mental health (n=65)		Depression (n=13)			
**App store category**										
	Education	1 (5)		0 (0)		0 (0)		1 (1)		
	Health and fitness	19 (95)		46 (71)		8 (62)		73 (74)		
	Lifestyle	0 (0)		5 (8)		0 (0)		5 (5)		
	Medical	0 (0)		14 (22)		5 (38)		19 (19)		
**App store rating**										
	3.6 star to 5 star	15 (75)		52 (80)		6 (46)		73 (74)		
	1 star to 3.5 star	0 (0)		7 (11)		2 (15)		9 (9)		
	No ratings	5 (25)		6 (9)		5 (38)		16 (16)		
**App cost**										
	Free	8 (40)		27 (42)		4 (31)		39 (40)		
	Free with in-app purchase	11 (55)		28 (43)		9 (69)		48 (49)		
	Paid	1 (5)		10 (15)		0 (0)		11 (11)		
**Language**										
	English	16 (80)		47 (72)		12 (92)		75 (77)		
	English and other languages	4 (20)		18 (28)		1 (8)		23 (23)		
**Target user of the app**										
	No target user	20 (100)		65 (100)		11 (85)		96 (98)		
	Youth (ages 12-18 years)	0 (0)		0 (0)		2 (15)		2 (2)		
**Psychotherapy modalities used by the app**										
	CBT^a^	8 (40)		31 (48)		9 (69)		48 (49)		
	CBT and other modalities	12 (60)		34 (52)		4 (31)		50 (51)		
**Number of evidence-based CBT techniques offered by the app**										
	0	5 (25)		4 (6)		0 (0)		9 (9)		
	1	3 (15)		6 (9)		1 (8)		10 (10)		
	2	11 (55)		25 (34)		2 (15)		38 (39)		
	3	1 (10)		10 (20)		2 (15)		13 (13)		
	4	0 (0)		16 (23)		8 (62)		24 (24)		
	5	0 (0)		4 (8)		0 (0)		4 (4)		
	6	0 (0)		0 (0)		0 (0)		0 (0)		
**Emergency contact information for users at risk of suicide**										
	Yes	2 (10)		23 (35)		8 (62)		33 (34)		
	No	18 (90)		42 (65)		5 (38)		65 (66)		
**Information related to COVID-19 pandemic**										
	Yes	0 (0)		16 (25)		1 (8)		17 (17)		
	No	20 (100)		49 (75)		12 (92)		81 (83)		
**App works as intended^b^**										
	Yes	18 (90)		62 (95)		12 (92)		92 (94)		
	No	2 (10)		3 (5)		1 (8)		6 (6)		

^a^CBT: cognitive behavioral therapy.

^b^Did not malfunction or crash during usage.

**Table 2:** List of countries where the self-guided cognitive behavioral therapy–based apps were developed (N=98).

Country	App category, n (%)				Total, n (%)
	Well-being (n=20)	Mental health (n=65)	Depression (n=13)		
Australia	0 (0)	4 (6)	2 (15)	6 (6)	
Bulgaria	2 (10)	0 (0)	0 (0)	2 (2)	
Canada	1 (5)	1 (2)	3 (23)	5 (5)	
Colombia	0 (0)	1 (2)	0 (0)	1 (1)	
Cyprus and Belarus	1 (5)	0 (0)	0 (0)	1 (1)	
Denmark	2 (10)	0 (0)	0 (0)	2 (2)	
Germany	0 (0)	4 (6)	0 (0)	4 (4)	
India	2 (10)	4 (6)	1 (8)	7 (7)	
Israel	0 (0)	2 (3)	0 (0)	2 (2)	
New Zealand	0 (0)	2 (3)	0 (0)	2 (2)	
Norway	0 (0)	2 (3)	1 (8)	3 (3)	
Poland	2 (10)	3 (5)	0 (0)	5 (5)	
Sweden	2 (10)	0 (0)	0 (0)	2 (2)	
The Netherlands	0 (0)	1 (2)	0 (0)	1 (1)	
United Kingdom	0 (0)	10 (15)	2 (15)	12 (12)	
United States	6 (30)	28 (43)	4 (31)	38 (39)	
Unknown	2 (10)	3 (5)	0 (0)	5 (5)	

A total of 25% (16/65) of mental health apps and 8% (1/13) of depression apps offered advice to manage the uncertainties and anxiety associated with COVID-19. In addition, 2 apps offered their subscription-only content for free to support users during the pandemic.

### CBT-Related Features

#### Overview

Apps included a wide variety of CBT-related features, from structured CBT modules resembling face-to-face interventions to journaling applications, presenting guided questionnaires loosely based on the cognitive model. Table 3 outlines the CBT-related features offered by the apps.

**Table 3:** Cognitive behavioral therapy–related techniques offered by the apps (N=98).

Feature			App category, n (%)							Total (N=98), n (%)
			Well-being (n=20)		Mental health (n=65)		Depression (n=13)			
**Evidence-based CBT^a^ techniques offered by the apps**										
	Psychoeducation	2 (10)		44 (68)		11 (85)		57 (58)		
	Behavioral activation	2 (10)		28 (43)		11 (85)		41 (42)		
	Cognitive restructuring	10 (50)		54 (83)		13 (100)		77 (79)		
	Problem solving	4 (20)		6 (9)		2 (15)		12 (12)		
	Relaxation	10 (50)		38 (38)		6 (46)		54 (55)		
	Exposure	1 (5)		4 (6)		0 (0)		5 (5)		
**Procedures related to the structure of CBT sessions**										
	Content offered in modules	2 (10)		12 (18)		8 (62)		22 (22)		
	Current mood monitoring	14 (70)		45 (69)		7 (54)		66 (67)		
	App administers screening questionnaire	0 (0)		21 (32)		11 (85)		32 (33)		
	Suicide risk management	2 (10)		23 (35)		9 (69)		34 (35)		
	Homework assignment	0 (0)		16 (25)		4 (31)		20 (20)		
	Therapeutic alliance	14 (70)		29 (45)		3 (23)		46 (47)		
	Strategies to cope after completing the modules	2 (10)		3 (5)		4 (31)		9 (9)		
**Other functionalities**										
	Journaling	3 (15)		7 (11)		0 (0)		10 (10)		
	Gratitude or positive thought records	2 (10)		10 (15)		0 (0)		12 (12)		
	Forums	2 (10)		4 (6)		0 (0)		6 (6)		
	Web-based games	1 (5)		4 (6)		0 (0)		6 (6)		
	Others	1 (5)		18 (28)		5 (38)		24 (25)		

^a^CBT: cognitive behavioral therapy.

#### Evidence-Based CBT Techniques

##### Overview

Most well-being apps (19/20, 95%) and over half of the mental health apps (35/65, 54%) offered up to two evidence-based CBT techniques, whereas most depression apps (10/13, 77%) offered three or four evidence-based techniques. Table 3 presents a summary of the CBT techniques assessed. A total of 25% (5/20) of well-being apps and 6% (4/65) of mental health apps offered questionnaires or journaling templates based on the cognitive model but no evidence-based techniques.

##### Psychoeducation

Psychoeducation offered by the apps was mostly related to aspects of CBT, that is, explanations about cognitive distortions assessed in the app, automatic thoughts, and the CBT model. Information about depression was found in over a third of mental health apps (15/44, 34%) and most of the depression apps (10/11, 91%).

##### Behavioral Activation

A total of 42% (41/98) of apps offered BA techniques or modules frequently assisting users to track (37/41, 90%) or schedule (30/41, 73%) their activities, whereas 56% (23/41) of apps suggested activities to engage in.

##### Cognitive Restructuring

Overall, 79% (77/98) of apps of the apps offered cognitive restructuring. Most apps (73/77, 95%) guided users to identify automatic thoughts, whereas approximately two-third of the apps offered guidance to reframe automatic thoughts (55/77, 71%) and identify the emotion linked to automatic thoughts (50/77, 65%) and/or cognitive distortions associated with automatic thoughts (48/77, 62%). Approximately two-third of the apps (50/77, 65%) obtained information using a template of follow-up questions similar to a thought record chart. A total of 6 apps (6/77, 8%) offered a noninteractive thought record template as their only feature.

##### Problem Solving

Problem-solving techniques or modules were offered by 12% (12/98) of apps, consisting of templates to plan a series of steps to solve a given problem.

##### Relaxation

A total of 54 apps offered relaxation modules. They consisted of mindfulness or meditation (33/54, 61%) apps or breathing exercise (31/54, 57%) apps. In total, 85% (46/54) of apps developed their own relaxation modules, whereas the rest offered links to other apps or websites.

##### Exposure Techniques for Comorbid Anxiety

In total, 5% (1/20) of well-being apps and 6% (4/65) of mental health apps included techniques to expose users to anxiety-provoking stimuli. A total of 3 apps requested the user to record the exposure exercises, whereas 2 apps offered a reward for completed tasks.

#### Procedures Related to the Structure of Face-to-Face CBT Sessions

##### Overview

A total of 22 apps offered content in structured modules. These modules resembled face-to-face CBT sessions in the 3 depression apps. Furthermore, 9 apps offered advice on how to cope at the end of the modules.

##### Mood Monitoring

A total of 66 apps inquired about users’ current mood, of which 20 (30%) apps queried about reasons for low or depressed mood. In addition, 32 mental health and depression apps administered a screening questionnaire, particularly the depression module of the Patient Health Questionnaire (PHQ; PHQ-9: 20/32, 63%; PHQ-8: 2/32, 6%) alone or accompanied by another questionnaire, including Generalized Anxiety Disorder-7 (13 apps), Work and Social Adjustment Scale (3 apps), and Depression, Anxiety, and Stress Scale-21 (1 app). Moreover, 6% (2/32) of apps administered only Depression, Anxiety, and Stress Scale-21, and 25% (8/32) of apps administered a nonvalidated questionnaire.

##### Suicide Risk Management

Just over a third (34/98, 35%) of apps acknowledged suicide risk associated with depression by listing crisis management resources or actively inquiring about suicide risk. A total of 3% (2/65) of mental health apps and 8% (1/13) of depression apps directly asked users about suicidal thoughts. Other apps, including 3% (2/65) of mental health and 31% (4/13) of depression apps passively assessed suicide risk through question 9 of the PHQ-9 (“thoughts that you would be better off dead or of hurting yourself in some way”). Although 4 apps responded to a positive answer to question 9 by offering access to crisis helplines, 2 apps did not actively respond to PHQ-9 results, instead offering a list of suicide prevention resources. A total of 6% (6/65) of mental health apps were conversational agents that responded to users’ suicidal thoughts by offering access to crisis helplines directly through the app. Finally, 10% (2/20) of well-being apps, 20% (13/65) of mental health apps, and 31% (4/13) of depression apps presented a list of crisis helpline numbers without assessing suicide risk.

##### Homework Assignment

A total of 20% (20/98) of apps offered users homework activities. In total, 70% (14/20) of apps assigned varied homework tasks, such as mood monitoring, reframing thoughts, BA, and relaxation exercises, whereas 30% (6/20) of apps consisted of a homework template to be completed between therapist visits.

##### Therapeutic Alliance

Overall, 46% (46/98) of apps included one or more engagement strategies comprising push notifications or reminders to access app features (31/46, 67%), offering feedback or encouraging messages (32/46, 70%) and/or awarding badges or other virtual rewards for completed activities (18/46, 39%). One conversational agent [66] was able to remember past conversations to further personalize the interaction.

##### Other Features

A total of 48% (47/98) of apps, including 45% (9/20) of well-being apps, 49% (32/65) of mental health apps, and 46% (6/13) of depression apps, also included one or more non–CBT-based features, particularly unstructured writing in the form of journals, gratitude, or positive thoughts; web-based games; and forums to connect to peers. A variety of other non–CBT-based functionalities were present in 1 or 2 assessed apps. These included inspiring quotes, visualization techniques, personality tests, access to self-help books, food records, management strategies for anxiety and panic attacks, safety plan templates, medication reminders, acceptance and self-compassion modules, music tracks, videos, physical exercise routines, and jokes.

### Technical Aspects and Quality Assurance

#### Overview

In general, the assessed apps were easy to use and provided a logical and simple layout to navigate. Most apps worked as intended, and only 6% (6/98) apps either crashed while in use or included nonfunctional features. None of the assessed apps included advertisements. A total of 46% (45/98) of apps allowed users to share data with health care providers, members of their support network, or other users of the app, using email or data syncing. Table 4 provides a summary of technical aspects and quality assurance of the apps.

**Table 4:** Technical features and quality assurance of included apps (N=98).

Feature				App category, n (%)							Total (N=98), n (%)
				Well-being (n=20)		Mental health (n=65)		Depression (n=13)			
**App credibility**											
	App content referenced or signed by the author			0 (0)		29 (45)		9 (69)		38 (39)	
	App includes a disclaimer that app information does not replace health care provider’s advice			5 (25)		41 (63)		9 (69)		55 (56)	
	**App development team mentioned in app content**										
		Government agency, academic institution, or nongovernment organization	2 (10)		4 (6)		0 (0)		6 (6)		
		Health care professional	1 (5)		36 (55)		11 (85)		48 (49)		
		Not declared	17 (85)		25 (38)		2 (15)		44 (45)		
**Data privacy**											
	Authentication required to access app			12 (60)		46 (71)		9 (69)		67 (68)	
	**App with a privacy policy^a^**			18 (90)		59 (91)		12 (92)		89 (91)	
		Presented before account creation	5 (6)		26 (29)		3 (25)		34 (35)		
		Explains how data are collected	17 (94)		58 (98)		12 (100)		87 (98)		
		Shares information with third-party providers	14 (78)		51 (86)		9 (75)		74 (83)		
		Shares contact details of Data Protection Officer	7 (35)		13 (22)		0 (0)		20 (22)		
	App allows users to share data			4 (20)		35 (59)		6 (50)		45 (51)	

^a^Percentage calculation was based on the number of apps with privacy policy (well-being apps: 18; mental health apps: 59; depression apps: 12; total: 89).

#### App Credibility

App content was referenced by 58% (57/98) of apps, including 10% (2/20) of well-being apps, 68% (44/65) of mental health apps, and 85% (11/13) of depression apps. Overall, just over half (54/98, 55%) of the apps were developed by academic institutions or included psychiatrists or psychologists in the development team. Disclaimers were present in 55 apps.

A total of 18 publications in peer-reviewed journals were available for 17% (17/98) of apps, including 25% (16/65) of mental health apps and 8% (1/13) of depression apps. A total of 33% (6/18) of papers included randomized controlled trials [67-72] reporting that apps or some specific app functions were effective in improving mood, anxiety, stress, or general well-being compared with active or waitlist controls. Other publications included pilot studies (3/18, 17%) [73-75], secondary data analysis (1/18, 6%) [76], and app usage data analyses (8/18, 44%) [77-84] to assess user engagement and/or app effectiveness in decreasing users’ symptoms.

#### Data Privacy and Security

Two-thirds (67/98, 68%) of the apps required authentication to access the app in the form of a password or less often a 4- to 6-digit numerical PIN. In addition, 49% (48/98) of apps allowed users to customize the privacy settings in the app.

Most apps (90/98, 92%) offered a privacy policy accessible from the app itself or through a link from the app store. The privacy policy was presented to users before account creation in only one-third of the apps (34/98, 35%). Most privacy policies (88/90, 98%) explained how users’ personal data were collected, and more than three-fourth of which (74/90, 82%) stated that they shared data with third parties, often service providers. Nonetheless, only a few apps mentioned the names of the service providers or explicitly stated the type of data shared with them. A total of 2 apps stated that they shared data with advertising companies. Just over half of the privacy policies (47/90, 52%) addressed the requirements of the General Data Protection Regulation mentioning the users’ right to have their data corrected or deleted, whereas only 24% (22/90) privacy policies included the contact details of a data protection officer.

## Discussion

### Principal Findings

Our systematic assessment of 98 Android (including 5 apps downloaded more than 1,000,000 times) and iOS self-guided CBT apps revealed a heterogeneous group offering a range of evidence-based [53,54] and non–evidence-based CBT techniques.

Only 4 mental health apps offered all five evidence-based CBT techniques. Depression apps consistently offered three to four techniques, whereas most well-being and mental health apps offered two evidence-based techniques, suggesting that only a few apps currently offer comprehensive, self-guided CBT programs that may benefit users who are unable to access face-to-face psychotherapy. Cognitive restructuring was the most common technique across all app categories. Psychoeducation and BA were offered by most depression apps and approximately half of mental health apps; however, they were seldomly included in well-being apps. In addition, well-being and mental health apps frequently offered relaxation and mindfulness, whereas less than half of depression apps did so.

Our study’s broad inclusion criteria aimed to resemble the options offered by app marketplaces when users search for a self-help app. Furthermore, classifying the apps into three distinct groups revealed that depression apps consistently offered more comprehensive programs, including at least three evidence-based techniques, whereas mental health and well-being apps were substantially less adherent. This finding differs from previous studies evaluating mental health apps [52,57,85,86] and, more specifically, CBT for depression [47,48] apps that consistently reported low adherence to evidence-based techniques. Huguet et al [47] evaluated 12 CBT and BA apps for depression available in Canada and reported a median adherence to core CBT criteria of 15%, with 2 apps adhering to 75% of the criteria. Stawarz et al [48] analyzed 31 CBT apps and reported that most apps included cognitive restructuring and offered one or two CBT techniques.

Only one-third of the assessed apps offered resources to address suicide risk, whereas only a minority actively assessed users’ suicide risk. App developers appear to disregard suicide risk when designing well-being and mental health apps, a worrying trend considering that most people dying by suicide are affected by a mental disorder [87,88].

Although most apps included a privacy policy mentioning how users’ personal data were used and shared, only a few provided sufficient details on the type of data shared or the companies with which the data were shared. Previous studies have shown that app developers often share user data with third parties, including Google and Facebook advertising [45,89], even when this is not stated in the privacy policy. Despite increasing concerns with regard to data privacy and security, apps still present considerable data management shortcomings, such as allowing third-party services to install pieces of code in the app to secretly access user data [90], as data sharing is a source of revenue, particularly for free apps. Notwithstanding the repeated calls for improvement, app development and publication processes remain unclear, allowing for loose interpretation or disregard of regulations. Further steps still need to be taken to ensure that user data are not misused, particularly highly sensitive data such as mental health–related information.

None of the assessed apps offered personalized content beyond including usernames or sex-appropriate pronouns when offering feedback or during dialogs with conversational agents. As a proxy to protect users’ privacy, apps did not ask personal questions beyond name, sex, or age, nor did they inquire about relevant medical history. Other engagement features, such as push notifications, reminders, and gamification, were found in less than half of the included apps. Although the role of the *bond* between users and interventions in self-guided digital interventions remains unclear, it appears that tailoring content, personalization, and interactive features such as reminders, positive feedback, and supporting social interactions with other app users improve engagement and adherence [55].

One of the advantages of digital technologies is the possibility of continuous updates and improvements on the basis of contextual challenges or new clinical guidelines. However, as only 17% (17/98) of the included apps included information on the impact of COVID-19 and containment measures on mental health [17], it can be assumed that digital mental health providers do not fully leverage available technological opportunities.

Publications in peer-reviewed journals were available for 17 apps, of which only 6 (35%) apps were evaluated using a randomized clinical trial, confirming the lack of evidence supporting the use of publicly available apps [91,92]. When evidence is available, clinical trials have consistently shown that apps improve mood in users affected by depression [67,68] during and shortly after the completion of the trial, even if high dropout rates limit the validity of the results [44].

This study, using broad inclusion criteria, showed a great variety of CBT-based apps, from simple journaling apps to structured modules mirroring traditional CBT. Although such extensive selection may positively affect a larger number of users, it might be cumbersome and frustrating for users that specifically require a more structured self-guided CBT program, potentially hampering access to self-help tools. Mental health apps follow a continuum from well-being and lifestyle apps for healthy users to apps offering self-guided psychotherapy as an adjuvant to ongoing traditional therapy or as the sole therapy for people with mental health disorders. As such, the current app store categories may not be suitable for mental health apps. We propose the creation of a new, encompassing mental health category to include all well-being and mental health specific apps with subcategories targeting different mental health disorders, therapy modalities, and user groups. In addition, a wider overhaul of health app development and publication processes is required.

### Strengths and Limitations of the Study

Our study had several strengths. Our process for searching, retrieving, and assessing included apps is grounded in systematic review methodology. The app search was implemented using the search engine of a commercial app database that allowed for a geographically unrestricted app search. Comprehensive assessment criteria were developed using reputable CBT manuals and a framework developed by our center to ensure comprehensive results.

However, this study has several limitations. Our search strategy included only CBT-related terms, potentially missing other apps offering CBT-based therapy for users with low mood or depression. We included apps offering self-guided CBT interventions, given the ethical limitations of using a simulated app persona when interacting with a health care provider, potentially excluding from our analysis more comprehensive apps. Apps linked to a specific provider or requiring an access code were also excluded from our study. Furthermore, the app store search was limited to only four categories with the maximum probability of retrieving relevant apps; therefore, we may have missed other apps outside these categories. Only apps in English were included in our study, potentially excluding relevant apps in other languages.

### Conclusions

Self-guided CBT-based apps available in app marketplaces offer a wide range of interventions; however, only approximately one-third, particularly depression apps, included comprehensive CBT programs. App developers’ access, use, and sharing of user data are unclear, raising concerns about the privacy and security of user data, and highlighting severe shortcomings in the governance of the health app market. Only a few apps offered suicide risk management resources or information on the current COVID-19 pandemic. The classification of mental health apps may benefit from the creation of a new mental health app category, including all well-being and specific disorder apps. To fulfill their potential, it is essential that self-guided CBT-based apps adhere to evidence-based clinical guidelines, be patient-centered, and offer enhanced and transparent data security measures.

## Appendix

Multimedia Appendix 1 Assessment criteria for cognitive behavioral therapy–related features.

Multimedia Appendix 2 Characteristics of included apps.

## Abbreviations

BA: behavioral activation
CBT: cognitive behavioral therapy
iCBT: internet-based cognitive behavioral therapy
PHQ: Patient Health Questionnaire
