# Supplementary material for: Self-guided Cognitive Behavioral Therapy Apps for Depression: Systematic Assessment of Features, Functionality, and Congruence With Evidence
Source: J Med Internet Res. 2021 Jul 30;23(7):e27619. doi: 10.2196/27619 (PMC8367167; doi:10.2196/27619)
Supplement: Multimedia Appendix 1 [file jmir_v23i7e27619_app1.docx]

**Supplementary Table 1**: CBT-related features assessment criteria

| 2-1 Patient Education | 2-1-1 | Does the app educate users on the symptoms and diagnosis of depression? | Yes/No | "Cognitive behavior therapy is educative… the nature and course of her disorder, ..." (Beck) (** The CBT education component is predominantly focused on ABC , i.e. teaching link between thoughts emotions and actions) |
| --- | --- | --- | --- | --- |
|  | 2-1-2 | Does the app educate users on the principles and use of Cognitive Behavioral Therapy? | Yes/No | "…about the process of cognitive behavior therapy…" (Beck) ** |
|  | 2-1-3 | Does the app explain the "cognitive model"? | Yes/No | "… and about the cognitive model (i.e., how her thoughts influence her emotions and behavior)…" (Beck) |
|  | 2-1-4 | Does the app explain what basic beliefs (core and intermediate beliefs) are? | Yes/No | "...core beliefs are enduring understandings so fundamental and deep that they often do not articulate them, even to themselves. The person regards these ideas as absolute truths—just the way things “are” "Educate patients about core beliefs in general and about their specific core beliefs; ..." (Beck) "… intermediate class of beliefs, which consists of (often unarticulated) attitudes, rules, and assumptions…" (Beck) |
|  | 2-1-5 | Does the app explain what automatic thoughts are? | Yes/No | "Automatic thoughts, the actual words or images that go through a person’s mind, are situation specific and may be considered the most superficial level of cognition." (Beck) |
|  | 2-1-6 | Does the app explain what cognitive distortions are? | Yes/No | "Patients tend to make consistent errors in their thinking. Often there is a systematic negative bias in the cognitive processing of patients who suffer from a psychiatric disorder…" (Beck) |
|  | 2-1-7 | Does the app explain how to use record forms (e.g. activity trackers, thought records, etc.)? | Yes/No | The Thought Record (TR), … is a worksheet that prompts patients to evaluate their automatic thoughts when they feel distressed (Beck) |
|  | 2-1-8 | Does the app stress the importance of homework in CBT? | Yes/No | Homework is an integral, not optional, part of cognitive behavior therapy (Beck) |
| 2-2 Behavioral Activation | 2-2-1 | Does the app guide users to overcome "depressive-passivity" and become more active? | Yes/No | "Behavioral activation is essential for most depressed patients. Many patients need only to be provided with a rationale, guidance in selecting and scheduling activities, and responses to predicted automatic thoughts that might interfere with implementing the activities or with gaining a sense of pleasure or mastery from them." (Beck) |
|  | 2-1-2 | Does the app guide users to track their daily routines/ activities? | Yes/No |  |
|  | 2-1-2-1 | If yes, how does the app do so? | App guides user with follow-up questions/ App provides a template (activity chart) for user to complete/ Other (please specify) |  |
|  | 2-1-3 | Does the app guide users to schedule tasks or fun or pleasurable activities? | Yes/No |  |
|  | 2-1-3-1 | If yes, how does the app do so? | App guides user with follow-up questions/ App provides a template for user to complete/ Other (please specify) |  |
|  | 2-1-4 | Does the app track completed activities? | Yes/No |  |
|  | 2-1-4-1 | If yes, how does the app do so? | App guides user with follow-up questions/ App provides a template for user to complete/ Other (please specify) |  |
|  | 2-1-5 | Does the app suggest fun or pleasurable activities for users to engage in? | Yes/No |  |
|  | 2-1-5-1 | If yes, how does the app do so? | App guides user with follow-up questions/ App provides a template for user to complete/ Other (please specify) |  |

**Supplementary Table 1**: CBT-related features assessment criteria (continued)

| 2-3 Cognitive Restructuring | 2-3-1 Automatic thoughts (AT) | 2-3-1-1 | Does the app guide users to identify their automatic thoughts? | Yes/ No/ NA | "Cognitive behavior therapy teaches patients to identify, evaluate, and respond to their dysfunctional thoughts and beliefs." "Learning to evaluate automatic thoughts is a skill..." (Beck) |
| --- | --- | --- | --- | --- | --- |
|  |  | 2-3-1-2 | If yes, how does the app do so? | App guides user with follow-up questions (e.g. *“What was going through your mind?”*)/ App provides a template for the user to complete (Thought record)/ Other (please specify) |  |
|  |  | 2-3-1-3 | Does the app enquire about specific situations that elicit the automatic thought? | Yes/No | "… a wide range of both external stimuli and internal experiences can give rise to automatic thoughts…" (Beck) |
|  |  | 2-3-1-4 | If yes, how does the app do so? | App guides user with follow-up questions/ App provides a template for the user to complete (Thought record)/ Other (please specify) |  |
|  |  | 2-3-1-5 | Does the app guide users to identify the emotion linked to their automatic thoughts? | Yes/No | "… how their underlying beliefs give rise to specific automatic thoughts in a specific situation, influencing their emotions and behavior." (Beck) |
|  |  | 2-3-1-6 | If yes, how does the app do so? | App guides user with follow-up questions/ App provides a template for user to complete (Thought record)/ Other (please specify) |  |
|  |  | 2-3-1-7 | Does the app guide users to identify the cognitive distortions associated to the automatic thoughts? | Yes/No | "It often helps to label distortions and to teach patients to do the same…" "You can also provide patients with a list of distortions…" (Beck) *See list of cognitive distortions in next sheet* |
|  |  | 2-3-1-8 | If yes, how does the app do so? | App guides user with follow-up questions/ App provides a template for user to complete (Thought record)/ Other (please specify) |  |
|  |  | 2-3-1-9 | Does the app guide user to come up with new explanations to the automatic thought? | Yes/No | "Therapists help patients identify key cognitions and adopt more realistic, adaptive perspectives, which leads patients to feel better emotionally, behave more functionally, and/or decrease their physiological arousal. They do so through the process of guided discovery, using questioning (often labeled or mislabeled as “Socratic questioning”) to evaluate their thinking (rather than persuasion, debate, or lecturing)" (Beck) |
|  |  | 2-3-1-10 | If yes, how does the app do so? | App guides user with follow-up questions (e.g. *What is the evidence that the AT is true? Not true?; Is there an alternative explanation?; What’s the worst that could happen? How could I cope?*/ App provides a template for user to complete (Thought record)/ Other (please specify) |  |
|  | 2-3-2 Thought diary (or thought record) | 2-3-2-1 | Does the app ask users to complete a thought diary (or thought record)? | Yes/No | "The Thought Record (TR), … is a worksheet that prompts patients to evaluate their automatic thoughts when they feel distressed…" The components of a thought record worksheet are: *Date/Time, Situation, Automatic Thought(s), Emotion(s), Adaptive Response (Cognitive Distortions), Outcome* |
|  |  | 2-3-2-2 | If yes, how does the app do so? | App guides the user with follow-up questions/ App provides a template for the user to complete/ Other (please specify) |  |
|  | 2-3-3 Core beliefs | 2-3-3-1 | Does the app differentiate between core beliefs and automatic negative thoughts? | Yes/No | "Cognitive behavior therapy teaches patients to identify, evaluate, and respond to their dysfunctional thoughts and beliefs" |
|  |  | 2-3-3-2 | Does the app guide user to identify their "core beliefs"? | Yes/No | "… you will teach patients the tools of identifying, evaluating, and adaptively responding to automatic thoughts and intermediate beliefs before using the same tools for core beliefs." (Beck) The components of a core belief worksheet are: *Evidence supporting new core belief/ Evidence supporting old core belief* |
|  |  | 2-3-3-3 | Does the app guide users to develop a new "core belief", more realistic, and functional? | Yes/No |  |
|  |  | 2-3-3-4 | If yes, how does the app do so? | App guides the user with follow-up questions/ App provides a template for the user to complete (e.g. Core Belief Worksheet) / App guides users through specific activities (e.g. acting “as if”, behavioral experiments)/ Other (please specify) |  |

**Supplementary Table 1**: CBT-related features assessment criteria (continued)

|  | 2-3-4 Behavioral experiments | 2-3-4-1 | Does the app challenge users' thoughts using behavioral experiments? | Yes/No | "Therapists also create experiences, called behavioral experiments, for patients to directly test their thinking" (Beck) |
| --- | --- | --- | --- | --- | --- |
| 2-4 Other cognitive techniques | 2-4-1 Problem Solving | 2-4-1-1 | Does the app guide users to problem-solve? | Yes/No | "Help alleviate their distress through a variety of techniques and problem solving." "behavioral and problem-solving techniques are essential, as are techniques from other orientations that are implemented within a cognitive framework." (Beck) |
|  |  | 2-4-1-2 | If yes, how does the app do so? | App guides the user with follow-up questions/ App provides a template for the user to complete/ Other (please specify) |  |
|  | 2-4-2 Relaxation | 2-4-2-1 | Does the app suggest users to practice relaxation techniques? | Yes/No | "Many patients benefit from learning relaxation techniques..." (Beck) |
|  |  | 2-4-2-2 | What kind of relaxation module does the app offer? | Breathing exercises/ Mindfulness/ Music therapy/ Exercise/ Imagery/ Audio/ Meditation/ Others (Please specify) |  |
|  |  | 2-4-2-3 | If yes, how does the app provides the relaxation module? | The module is provided by the app/ The app provides a link to a third party app or website/ Other (please specify) |  |
|  | 2-4-3 Exposure | 2-4-3-1 | Does the app challenge users to engage in activities that generate anxiety? | Yes/No | "When patients are anxious and significantly avoidant, you will provide a strong rationale for exposing themselves to feared situations" (Beck) |
|  |  | 2-4-3-2 | If yes, how does the app do so? | App offer rewards for completed tasks/ App request user to record completed tasks/ Other (please specify) |  |
| 2-6 CBT sessions structure | 2-6-1 History taking | 2-6-1-1 | Does the app enquire about what triggered current episode of depression? (e.g. actual or perceived interpersonal losses, family discord, domestic violence, past or current sexual or physical abuse or neglect) | Yes/No | "You will need to know about many areas of the patient’s current and past experience to develop a sound treatment plan (across sessions), plan treatment within sessions, develop a good therapeutic relationship, guide the patient in setting goals, and generally carry out effective treatment" (Beck) |
|  |  | 2-6-1-2 | Does the app enquire on the presence and quality of the user's support network? | Yes/No |  |
|  | 2-6-2 Structured sessions | 2-6-2-1 | Do the app modules follow a similar structure? | Yes/No | "A major goal of treatment is to make the process of therapy understandable to you and the patient… Adhering to a standard format (as well as teaching the tools of therapy to the patient) facilitates these objectives" (Beck) |
|  |  | 2-6-2-2 | Do the modules follow a face-to-face CBT session structure? (Do a mood check/ Set the agenda/ Obtain an update/ Review homework/ Work on a specific problem and teach cognitive behavior therapy skills/ Provide a summary/ Set new homework) | Yes/No |  |
|  | 2-6-3 Standardized monitoring | 2-6-3-1 | Does the app enquire about the user’s current mood? | Yes/No | "The mood check is usually brief. It helps you and patients keep track of how they are progressing…" (Beck) |
|  |  | 2-6-3-2 | Does the app administer a questionnaire to assess risk and/or severity of depression, anxiety or other mental disorder? | Yes/No | "… review the symptom checklists she filled out just prior to the session…" (Beck) |
|  |  | 2-6-3-3 | If yes, which questionnaire does the app administer? | Free text |  |
|  |  | 2-6-3-4 | Does the app ask for reasons of user’s low or anxious mood? | Yes/No | "...you aim to obtain a clear picture of situations that are distressing to patients…" (Beck) |
|  | 2-6-4 Goal setting | 2-6-5-1 | Does the app allow users to set goals on what they would like to work on? | Yes/No | "Setting goals and relating a treatment plan help provide patients with hope." (Beck) |
|  |  | 2-6-5-2 | If yes, how does the app do so? | App guides the user with follow-up questions/ App provides a template for the user to complete/ Other (please specify) |  |
|  | 2-6-5 Homework | 2-6-6-1 | Does the app assign the user homework activities? | Yes/No | "Homework is an integral, not optional, part of cognitive behavioral therapy" (Beck) |
|  |  | 2-6-6-2 | If yes, what kind of homework does the app offer? | Behavioral activation/ Behavioral experiments/ Monitoring automatic thoughts/ Problem solving/ Bibliotherapy/ Worksheets/ Others (Please specify) |  |

**Supplementary Table 1**: CBT-related features assessment criteria (continued)

|  | 2-6-6 Ending therapy | 2-6-6-1 | Does the app offer the user strategies to cope with low mood after completing the modules? | Yes/No | "You will begin to prepare patients for termination and relapse even in the initial session, as you tell them that your goal is to make treatment as time limited as possible, with the aim of helping them become their own therapist" (Beck) |
| --- | --- | --- | --- | --- | --- |
|  |  | 2-6-6-2 | If yes, what strategies do the app offer? | App review learnt skills/ App lists action plans for the foreseeable future problems based on the skills learnt/ Other (please specify) |  |
|  | 2-6-7 User engagement (Therapeutic alliance) | 2-6-7-1 | Does the app actively engage users to continue using the app over time? | Yes/No | "you need to engage patients so they will return to treatment for the next session." (Beck)  To test user engagement we will continuously use the app for 2 weeks |
|  |  | 2-6-7-2 | If yes, how does the app do so? | Push notifications/ Email/ Text messaging/ Other (please specify) |  |
|  |  | 2-6-7-3 | Does the app encourage users when completing a task? | Yes/ No/ NA |  |
|  |  | 2-6-7-4 | If yes, how does the app do so? | Positive message/ Feedback on completed task/ Share on social media/ Other (please specify) |  |
|  |  | 2-6-7-5 | Does the app encourage users when attaining a goal? | Yes/ No/ NA |  |
|  |  | 2-6-7-6 | If yes, how does the app do so? | Positive message/ Feedback on completed task/ Share on social media/ Other (please specify) |  |
| 2-7 Others | 2-7-1 Access to professional advice | 2-7-1-1 | Does the app include a health professional who is accessible to the user (without users inputting any health provider/ health professional information)? | Yes/No | Desirable functionality for added support |
|  |  | 2-7-1-2 | Is access to a health professional provided…? | Free of charge/ Requires in-app payment (please include currency and value as referred in the app) |  |
|  | 2-7-2 Suicide prevention | 2-7-2-1 | Does the app assess users’ suicidality? | Yes/No | "It is also critical to determine the degree to which patients might be suicidal" (Beck) |
|  | 2-7-3 Other modules | 2-7-3-1 | Does the app offer other functionalities to users? | Yes/No |  |
|  |  | 2-7-3-2 | If yes, what functionalities does the app offer? | Free text |  |
|  |  | 2-7-3-3 | Does the app provides information about mental health issues during COVID-19 pandemic? | Yes/No |  |
