# Supplementary material for: Self-guided Cognitive Behavioral Therapy Apps for Depression: Systematic Assessment of Features, Functionality, and Congruence With Evidence
Source: J Med Internet Res. 2021 Jul 30;23(7):e27619. doi: 10.2196/27619 (PMC8367167; doi:10.2196/27619)
Supplement: Multimedia Appendix 2 [file jmir_v23i7e27619_app2.docx]

**Supplementary Table 2**: Characteristics of included apps

| **App characteristics** | | | | **App functionalities** | | | | | | | | | | | | |
| --- | --- | --- | --- | --- | --- | --- | --- | --- | --- | --- | --- | --- | --- | --- | --- | --- |
| **App name** | **Platform** | **App category** | **HCP/ Acad Inst involved/**  **Country** | **Evidence-based CBT techniques** | | | | | | **Procedures related to the structure of CBT sessions** | | | | | | **Others** |
|  |  |  |  | **Psychoeducation** | **Behavioral activation** | **Cognitive restructuring** | **Problem solving** | **Relaxation** | **Exposure techniques** | **Content offered in modules** | **Mood monitoring** | **Suicide risk management** | **Homework assignment** | **“Therapeutic alliance”** | **End-of-therapy coping strategies** | **COVID-19 information** |
| 29k: Grow, with others | Android | Wellbeing | Acad Inst  Sweden | No | No | No | No | Yes | No | Yes | No | Yes | No | Yes | Yes | No |
| ABC-Schema | Android | Mental Health | No  Netherladns | No | No | Yes | No | No | No | No | No | No | No | No | No | No |
| Alegrify | Android | Wellbeing | No  Unknown | No | No | Yes | No | No | No | No | Yes | No | No | No | No | No |
| Arya - Mood Tracker & Activity Planner | Android | Mental Health | HCP  Germany | No | Yes | No | No | Yes | No | No | Yes | No | Yes | Yes | No | No |
| Calmer Sea: Digital therapy | Android | Mental Health | No  UK | No | No | No | No | Yes | No | No | Yes | No | No | No | No | No |
| Catch It | Android | Mental Health | Acad. Inst.  UK | Yes | No | Yes | No | No | No | No | No | No | Yes | No | No | No |
| CBT Diary | Android | Mental Health | No  Poland | No | No | Yes | No | No | No | No | No | No | No | No | No | No |
| CBT Mental Health Application | Android | Mental Health | No  Canada | Yes | No | Yes | No | Yes | No | No | Yes | Yes | No | No | No | No |
| CBT Thought Diary | Android | Mental Health | HCP  USA | Yes | No | Yes | No | No | No | No | No | No | Yes | No | No | No |
| CBT Tools for Healthy Living Self-help Diary | Android | Mental Health | HCP  USA | Yes | Yes | Yes | No | Yes | No | No | Yes | No | No | Yes | No | No |
| CBTwithyou | Android | Mental Health | HCP  Israel | Yes | No | Yes | No | No | No | No | No | No | No | No | No | No |
| Cognitive Behavioural Therapy (CBT Tools) | Android | Mental Health | No  UK | Yes | Yes | Yes | No | No | No | No | Yes | No | No | No | No | No |
| eGuru Depression/ eGuru Mood Diary/ eGuru Thought Diary | Android | Depression | Yes  Canada | No | Yes | Yes | No | No | No | No | Yes | No | No | No | No | No |
| ezeCBT - (CBT - Cognitive Behavioral Therapy) | Android | Mental Health | No  Unknown | Yes | No | Yes | No | No | No | No | Yes | No | No | No | No | No |
| feel better - Mood & CBT therapy to manifest goals | Android | Mental Health | No  USA | No | No | No | No | No | No | No | Yes | No | No | Yes | No | No |
| HCP: Healthcare provider; Acad. Inst: Academic Institution | | | | | |  |  |  |  |  |  |  |  |  |  |  |

**Supplementary Table 2**: Characteristics of included apps (continued)

| **App characteristics** | | | | **App functionalities** | | | | | | | | | | | | |
| --- | --- | --- | --- | --- | --- | --- | --- | --- | --- | --- | --- | --- | --- | --- | --- | --- |
| **App name** | **Platform** | **App category** | **HCP/ Acad. Institutions involved/**  **Country** | **Evidence-based CBT techniques** | | | | | | **Procedures related to the structure of CBT sessions** | | | | | | **Others** |
|  |  |  |  | **Psychoeducation** | **Behavioral activation** | **Cognitive restructuring** | **Problem solving** | **Relaxation** | **Exposure techniques** | **Content offered in modules** | **Mood monitoring** | **Suicide risk management** | **Homework assignment** | **“Therapeutic alliance”** | **End-of-therapy coping strategies** | **COVID-19 information** |
| Happify | Android | Mental Health | HCP  USA | Yes | Yes | Yes | No | Yes | No | Yes | No | No | Yes | Yes | No | Yes |
| Happyso | Android | Wellbeing | No  USA | No | No | Yes | No | Yes | No | No | Yes | No | No | Yes | No | No |
| HSpot: Emotional Wellbeing | Android | Wellbeing | Unknown  India | No | No | No | Yes | Yes | No | No | Yes | No | No | No | No | No |
| InnerHour: Calm,Sleep,Depression & Anxiety Therapy | Android | Mental Health | HCP  India | Yes | Yes | Yes | No | Yes | No | Yes | Yes | No | No | No | No | Yes |
| Lmtls for Stress & Anxiety | Android | Wellbeing | HCP  Canada | No | No | Yes | No | Yes | No | No | Yes | No | No | No | No | No |
| Marvin: Mental Health Chat | Android | Mental Health | No  USA | No | No | No | No | No | No | No | Yes | No | No | No | No | No |
| Mind+ | Android | Mental Health | Unknown  Colombia | Yes | No | Yes | No | No | No | Yes | Yes | No | No | No | No | No |
| Mindfit - stop negative thinking | Android | Mental Health | HCP  Norway | No | No | Yes | No | Yes | No | No | No | No | No | Yes | No | No |
| Moodfit - Stress & Anxiety | Android | Mental Health | No  USA | Yes | Yes | Yes | No | Yes | No | No | Yes | Yes | No | Yes | No | Yes |
| MoodMission - Cope with Stress, Moods & Anxiety | Android | Mental Health | HCP  Australia | No | Yes | No | No | Yes | Yes | Yes | Yes | No | No | No | No | No |
| Moodpath - Depression & Anxiety Test | Android | Mental Health | HCP  Germany | Yes | No | Yes | No | Yes | No | Yes | Yes | Yes | Yes | Yes | No | Yes |
| MoodSpace - Stress, anxiety, & low mood self-help | Android | Mental Health | No  UK | Yes | Yes | Yes | No | Yes | No | No | No | No | Yes | No | No | No (offer free access to paid content) |
| MoodTools - Depression Aid | Android | Depression | HCP  USA | Yes | Yes | Yes | No | Yes | No | No | No | Yes | No | No | No | No |
| Overcoming | Android | Mental Health | No  UK | Yes | Yes | Yes | No | No | No | No | Yes | No | No | No | No | No |
| Overcoming Depression | Android | Depression | No  UK | Yes | Yes | Yes | No | No | No | No | Yes | No | No | No | No | No |
| HCP: Healthcare provider; Acad. Inst: Academic Institution | | | | | |  |  |  |  |  |  |  |  |  |  |  |

**Supplementary Table 2**: Characteristics of included apps (continued)

| **App characteristics** | | | | **App functionalities** | | | | | | | | | | | | |
| --- | --- | --- | --- | --- | --- | --- | --- | --- | --- | --- | --- | --- | --- | --- | --- | --- |
| **App name** | **Platform** | **App category** | **HCP/ Acad. Institutions involved/**  **Country** | **Evidence-based CBT techniques** | | | | | | **Procedures related to the structure of CBT sessions** | | | | | | **Others** |
|  |  |  |  | **Psychoeducation** | **Behavioral activation** | **Cognitive restructuring** | **Problem solving** | **Relaxation** | **Exposure techniques** | **Content offered in modules** | **Mood monitoring** | **Suicide risk management** | **Homework assignment** | **“Therapeutic alliance”** | **End-of-therapy coping strategies** | **COVID-19 information** |
| Psychiatry Pro-Diagnosis,Info,Treatment,CBT & DBT | Android | Mental Health | HCP  Unknown | Yes | No | Yes | No | No | No | No | Yes | Yes | No | No | No | No |
| PsychStar | Android | Depression | HCP  Australia | Yes | Yes | Yes | No | Yes | No | No | No | Yes | No | No | No | No |
| PUSH-D | Android | Depression | HCP  India | Yes | Yes | Yes | No | Yes | No | Yes | Yes | Yes | Yes | No | Yes | No |
| Reflect - Journal, Daily guided self reflection | Android | Wellbeing | No  Bulgaria | No | No | No | No | No | No | No | Yes | No | No | Yes | No | No |
| Reflectly - Journal / Diary | Android | Wellbeing | No  Denmark | No | No | No | No | No | No | No | Yes | No | No | Yes | No | No |
| Sane - Guided Journaling & Mindfulness | Android | Mental Health | No  Unknown | No | No | No | No | No | No | No | Yes | No | No | No | No | No |
| Sanvello - Stress & Anxiety Help | Android | Mental Health | HCP  USA | Yes | Yes | Yes | No | Yes | No | Yes | Yes | Yes | Yes | Yes | Yes | Yes |
| Serenity: mental health, self-care & anxiety CBT | Android | Mental Health | HCP  USA | No | No | Yes | No | Yes | No | No | Yes | Yes | No | No | No | Yes |
| Tappily | Android | Wellbeing | No  USA | No | No | Yes | Yes | No | No | No | No | No | No | No | No | No |
| Therapify | Android | Mental Health | HCP  Poland | Yes | Yes | Yes | No | No | No | No | Yes | No | Yes | No | No | No |
| Thinkladder - Self-awareness & Mental Wellness | Android | Mental Health | Unknown  New Zealand | No | No | Yes | No | No | No | No | No | No | No | No | No | Yes |
| Thought Challenger | Android | Mental Health | Acad. Inst.  USA | No | No | Yes | No | No | No | No | No | No | No | Yes | No | No |
| Thoughts - CBT trainer and thought diary | Android | Mental Health | HCP  India | Yes | No | Yes | No | No | No | No | No | No | No | No | No | No |
| UpLift for Depression | Android | Depression | HCP  USA | Yes | Yes | Yes | Yes | No | No | Yes | Yes | Yes | Yes | Yes | Yes | No |
| What's Up? - A Mental Health App | Android | Mental Health | HCP  Australia | Yes | Yes | Yes | No | Yes | No | No | Yes | Yes | No | No | No | No |
| HCP: Healthcare provider; Acad. Inst: Academic Institution | | | | | |  |  |  |  |  |  |  |  |  |  |  |

**Supplementary Table 2**: Characteristics of included apps (continued)

| **App characteristics** | | | | **App functionalities** | | | | | | | | | | | | |
| --- | --- | --- | --- | --- | --- | --- | --- | --- | --- | --- | --- | --- | --- | --- | --- | --- |
| **App name** | **Platform** | **App category** | **HCP/ Acad. Institutions involved/**  **Country** | **Evidence-based CBT techniques** | | | | | | **Procedures related to the structure of CBT sessions** | | | | | | **Others** |
|  |  |  |  | **Psychoeducation** | **Behavioral activation** | **Cognitive restructuring** | **Problem solving** | **Relaxation** | **Exposure techniques** | **Content offered in modules** | **Mood monitoring** | **Suicide risk management** | **Homework assignment** | **“Therapeutic alliance”** | **End-of-therapy coping strategies** | **COVID-19 information** |
| Woebot: Your Self-Care Expert | Android | Mental Health | HCP  USA | Yes | Yes | Yes | No | Yes | No | No | Yes | Yes | No | Yes | No | Yes |
| Wysa: stress, depression & anxiety therapy chatbot | Android | Mental Health | HCP  USA | Yes | No | Yes | Yes | Yes | No | No | Yes | Yes | No | Yes | No | No |
| Youper - Emotional Health | Android | Mental Health | HCP  USA | Yes | Yes | Yes | Yes | Yes | No | No | Yes | Yes | No | Yes | No | No |
| 29k: Find Yourself & Others | iOS | Wellbeing | Acad. Inst.  Sweden | No | No | No | No | Yes | No | Yes | No | Yes | No | Yes | Yes | No |
| Am I? Behavioural Experiment | iOS | Depression | HCP  Canada | No | No | Yes | No | No | No | No | No | No | No | No | No | No |
| Am I? My Thought Journal | iOS | Depression | HCP  Canada | Yes | No | Yes | No | No | No | No | No | Yes | No | No | No | No |
| Arya - Activity Planner | iOS | Mental Health | HCP  Germany | No | Yes | No | No | Yes | No | No | Yes | No | Yes | Yes | No | No |
| Bloom: Self-Guided Therapy CBT | iOS | Mental Health | No  USA | Yes | No | Yes | No | Yes | No | Yes | Yes | Yes | No | Yes | No | No (offer free access to paid content) |
| Bold: CBT Journal | iOS | Wellbeing | No  USA | Yes | No | Yes | No | No | No | No | No | No | No | Yes | No | No |
| Braive: Mental Health Help | iOS | Depression | HCP  Norway | Yes | Yes | Yes | No | Yes | No | Yes | Yes | Yes | Yes | Yes | Yes | Yes |
| Breeze: mood tracker, diary | iOS | Wellbeing | No  Cyprus/Belarus | No | No | No | No | No | No | No | Yes | No | No | Yes | No | No |
| Calmer Sea: Digital therapy | iOS | Mental Health | No  UK | No | No | No | No | Yes | No | No | Yes | No | No | No | No | No |
| Catch It – Make sense of moods | iOS | Mental Health | Acad. Inst.  UK | Yes | No | Yes | No | No | No | No | No | No | Yes | No | No | No |
| CBT Companion | iOS | Mental Health | No  USA | Yes | Yes | Yes | No | Yes | Yes | No | Yes | No | No | Yes | No | No |
| CBT Thought Diary | iOS | Mental Health | HCP  USA | Yes | No | Yes | No | No | No | No | No | No | Yes | No | No | No |
| CBT Tools | iOS | Mental Health | No  UK | Yes | Yes | Yes | No | No | No | No | No | No | No | No | No | No |
| HCP: Healthcare provider; Acad. Inst: Academic Institution | | | | | |  |  |  |  |  |  |  |  |  |  |  |

**Supplementary Table 2**: Characteristics of included apps (continued)

| **App characteristics** | | | | **App functionalities** | | | | | | | | | | | | |
| --- | --- | --- | --- | --- | --- | --- | --- | --- | --- | --- | --- | --- | --- | --- | --- | --- |
| **App name** | **Platform** | **App category** | **HCP/ Acad. Institutions involved/**  **Country** | **Evidence-based CBT techniques** | | | | | | **Procedures related to the structure of CBT sessions** | | | | | | **Others** |
|  |  |  |  | **Psychoeducation** | **Behavioral activation** | **Cognitive restructuring** | **Problem solving** | **Relaxation** | **Exposure techniques** | **Content offered in modules** | **Mood monitoring** | **Suicide risk management** | **Homework assignment** | **“Therapeutic alliance”** | **End-of-therapy coping strategies** | **COVID-19 information** |
| CBTwithyou: CBT with you | iOS | Mental Health | HCP  Israel | Yes | No | Yes | No | No | No | No | No | No | No | No | No | No |
| Enlighten | iOS | Mental Health | No  USA | No | No | Yes | No | Yes | No | No | Yes | Yes | No | No | No | Yes |
| feelbetter – Moods and goals | iOS | Mental Health | No  USA | No | No | No | No | No | No | No | Yes | No | No | Yes | No | No |
| FelicityCBT | iOS | Mental Health | Acad. Inst.  USA | Yes | No | Yes | No | No | No | No | Yes | No | No | No | No | No |
| Happier You | iOS | Mental Health | HCP  UK | Yes | Yes | Yes | Yes | Yes | No | Yes | Yes | Yes | Yes | No | Yes | No |
| Happify: for Stress & Worry | iOS | Mental Health | HCP  USA | Yes | Yes | Yes | No | Yes | No | Yes | No | No | Yes | Yes | No | Yes |
| Happyso | iOS | Wellbeing | No  USA | No | No | Yes | No | Yes | No | No | Yes | No | No | Yes | No | No |
| HSpot: Emotional Wellbeing | iOS | Wellbeing | No  India | No | No | No | Yes | Yes | No | No | Yes | No | No | No | No | No |
| Icoachi: self-care & self-love | iOS | Wellbeing | No  Poland | No | Yes | Yes | No | Yes | No | No | Yes | No | No | Yes | No | No |
| Kintsugi | iOS | Mental Health | HCP  USA | Yes | No | No | No | Yes | No | No | Yes | Yes | No | Yes | No | No |
| Mindfit stop negative thoughts | iOS | Mental Health | HCP  Norway | Yes | No | Yes | No | Yes | No | No | No | No | No | Yes | No | No |
| Mood App: Journal | iOS | Mental Health | No  USA | No | No | Yes | No | Yes | No | No | Yes | No | No | Yes | No | No |
| Moodfit – Shape up your mood | iOS | Mental Health | No  USA | Yes | Yes | Yes | No | Yes | No | No | Yes | Yes | No | Yes | No | Yes |
| Mooditude – Mood tracker | iOS | Mental Health | No  USA | Yes | Yes | Yes | Yes | Yes | No | No | Yes | Yes | No | Yes | No | No |
| MoodKit | iOS | Mental Health | No  USA | Yes | Yes | Yes | No | No | No | No | Yes | No | No | Yes | No | No |
| MoodMission | iOS | Mental Health | HCP  Australia | No | Yes | No | No | Yes | Yes | Yes | Yes | No | No | No | No | No |
| Moodnotes | iOS | Wellbeing | No  USA | Yes | No | Yes | No | No | No | No | Yes | No | No | Yes | No | No |
| HCP: Healthcare provider; Acad. Inst: Academic Institution | | | | |  |  |  |  |  |  |  |  |  |  |  |  |

**Supplementary Table 2**: Characteristics of included apps (continued)

| **App characteristics** | | | | **App functionalities** | | | | | | | | | | | | |
| --- | --- | --- | --- | --- | --- | --- | --- | --- | --- | --- | --- | --- | --- | --- | --- | --- |
| **App name** | **Platform** | **App category** | **HCP/ Acad. Institutions involved/**  **Country** | **Evidence-based CBT techniques** | | | | | | **Procedures related to the structure of CBT sessions** | | | | | | **Others** |
|  |  |  |  | **Psychoeducation** | **Behavioral activation** | **Cognitive restructuring** | **Problem solving** | **Relaxation** | **Exposure techniques** | **Content offered in modules** | **Mood monitoring** | **Suicide risk management** | **Homework assignment** | **“Therapeutic alliance”** | **End-of-therapy coping strategies** | **COVID-19 information** |
| Moodpath: Depression & Anxiety | iOS | Mental Health | HCP  Germany | Yes | No | Yes | No | Yes | No | Yes | Yes | Yes | Yes | Yes | No | Yes |
| MoodTools: Depression Aid | iOS | Depression | HCP  USA | Yes | Yes | Yes | No | Yes | No | No | No | Yes | No | No | No | No |
| Overcoming-Depression | iOS | Depression | No  UK | Yes | Yes | Yes | No | No | No | No | Yes | No | No | No | No | No |
| Pocket CBT | iOS | Mental Health | HCP  UK | Yes | No | Yes | No | No | No | No | No | No | No | No | No | No |
| PsychStar | iOS | Depression | HCP  Australia | Yes | Yes | Yes | No | Yes | No | No | No | Yes | No | No | No | No |
| Quirk CBT | iOS | Mental Health | HCP  USA | Yes | No | Yes | No | No | Yes | No | No | No | No | Yes | No | No |
| Reflect - guided daily journal | iOS | Wellbeing | No  Bulgaria | No | No | No | No | No | No | No | Yes | No | No | Yes | No | No |
| Reflectly | iOS | Wellbeing | No  Denmark | No | No | No | No | No | No | No | Yes | No | No | Yes | No | No |
| Sanvello: Stress & Anxiety Help | iOS | Mental Health | HCP  USA | Yes | Yes | Yes | No | Yes | No | Yes | Yes | Yes | Yes | Yes | Yes | Yes |
| Serenity: Mental Health CBT | iOS | Mental Health | HCP  USA | No | No | Yes | No | Yes | No | No | Yes | Yes | No | No | No | Yes |
| stoic. mental health training | iOS | Wellbeing | No  Poland | No | No | Yes | No | Yes | Yes | No | Yes | No | No | Yes | No | No |
| Tappily | iOS | Wellbeing | No  USA | No | No | Yes | Yes | No | No | No | No | No | No | Yes | No | No |
| Therapify | iOS | Mental Health | HCP  Poland | No | Yes | Yes | No | No | No | No | Yes | No | Yes | No | No | No |
| Thinkladder - Self-awareness & Mental Wellness | iOS | Mental Health | Unknown  New Zealand | No | No | Yes | No | Yes | No | No | No | No | No | No | No | Yes |
| Think-Ups | iOS | Wellbeing | No  Unknown | No | Yes | No | No | Yes | No | No | No | No | No | No | No | No |
| UpLift for Depression | iOS | Depression | HCP  USA | Yes | Yes | Yes | Yes | No | No | Yes | Yes | Yes | Yes | Yes | Yes | No |
| What’s Up? – A Mental Health App | iOS | Mental Health | HCP  Australia | Yes | Yes | Yes | No | Yes | No | No | Yes | Yes | No | No | No | No |
| HCP: Healthcare provider; Acad. Inst: Academic Institution | | | | | |  |  |  |  |  |  |  |  |  |  |  |

**Supplementary Table 2**: Characteristics of included apps (continued)

| **App characteristics** | | | | **App functionalities** | | | | | | | | | | | | |
| --- | --- | --- | --- | --- | --- | --- | --- | --- | --- | --- | --- | --- | --- | --- | --- | --- |
| **App name** | **Platform** | **App category** | **HCP/ Acad. Institutions involved/**  **Country** | **Evidence-based CBT techniques** | | | | | | **Procedures related to the structure of CBT sessions** | | | | | | **Others** |
|  |  |  |  | **Psychoeducation** | **Behavioral activation** | **Cognitive restructuring** | **Problem solving** | **Relaxation** | **Exposure techniques** | **Content offered in modules** | **Mood monitoring** | **Suicide risk management** | **Homework assignment** | **“Therapeutic alliance”** | **End-of-therapy coping strategies** | **COVID-19 information** |
| Woebot - Your Self-Care Expert | iOS | Mental Health | HCP  USA | Yes | Yes | Yes | No | Yes | No | No | Yes | Yes | No | Yes | No | Yes |
| Wysa: Mental Health Support | iOS | Mental Health | HCP  USA | Yes | No | Yes | Yes | Yes | No | No | Yes | Yes | No | Yes | No | No |
| Youper | iOS | Mental Health | HCP  USA | Yes | Yes | Yes | Yes | Yes | No | No | Yes | Yes | No | Yes | No | No |
| HCP: Healthcare provider; Acad. Inst: Academic Institution | | | | | |  |  |  |  |  |  |  |  |  |  |  |
